# Distribution, mobility, and anchoring of lignin-related oxidative enzymes in Arabidopsis secondary cell walls

**DOI:** 10.1093/jxb/ery067

**Published:** 2018-02-22

**Authors:** Eva Yi Chou, Mathias Schuetz, Natalie Hoffmann, Yoichiro Watanabe, Richard Sibout, A Lacey Samuels

**Affiliations:** 1 Department of Botany, University of British Columbia, Vancouver, BC, Canada; 2 Institut Jean-Pierre Bourgin, UMR 1318, INRA, AgroParisTech, CNRS, Université Paris-Saclay, Versailles Cedex, France

**Keywords:** Fibers, laccases, lignin, peroxidases, secondary cell wall, xylem vessels

## Abstract

Lignin is an important phenolic biopolymer that provides strength and rigidity to the secondary cell walls of tracheary elements, sclereids, and fibers in vascular plants. Lignin precursors, called monolignols, are synthesized in the cell and exported to the cell wall where they are polymerized into lignin by oxidative enzymes such as laccases and peroxidases. In *Arabidopsis thaliana*, a peroxidase (PRX64) and laccase (LAC4) are shown to localize differently within cell wall domains in interfascicular fibers: PRX64 localizes to the middle lamella whereas LAC4 localizes throughout the secondary cell wall layers. Similarly, laccases localized to, and are responsible for, the helical depositions of lignin in protoxylem tracheary elements. In addition, we tested the mobility of laccases in the cell wall using fluorescence recovery after photobleaching. mCHERRY-tagged LAC4 was immobile in secondary cell wall domains, but mobile in the primary cell wall when ectopically expressed. A small secreted red fluorescent protein (sec-mCHERRY) was engineered as a control and was found to be mobile in both the primary and secondary cell walls. Unlike sec-mCHERRY, the tight anchoring of LAC4 to secondary cell wall domains indicated that it cannot be remobilized once secreted, and this anchoring underlies the spatial control of lignification.

## Teaser

Laccases and peroxidases localize to different wall domains in Arabidopsis stems. These enzymes are tightly anchored in the secondary cell wall, providing a mechanism for spatial control of lignification.

## Introduction

The invasion of terrestrial habitats by plants was made possible by the evolution of specialized cell types that deposit a thick secondary cell wall, which is essential for water/nutrient transport and structural support (Fukuda, 1996). Secondary cell walls are strong because of the architecture imparted by cellulose, hemicelluloses, and lignin. Abundant cellulose microfibrils are rapidly deposited by dense arrays of plasma membrane-bound cellulose synthase enzymes (CesAs) (Watanabe *et al.*, 2015; Li *et al.*, 2016). These are non-covalently linked to specific hemicelluloses, typically with mannan and xylan polysaccharides backbones (Rennie and Scheller, 2014). In Arabidopsis, the major hemicellulose in secondary cell walls is glucoronoxylan, and NMR demonstrates that the xylan is in close physical proximity to the cellulose microfibrils (Simmons *et al.*, 2016).

Lignin is formed from monolignols, which are derived from phenylalanine in the cytoplasm, before being transferred to the cell wall (Barros *et al.*, 2015). In the cell wall, the monolignols are oxidized by peroxidases and/or laccases to form monolignol radicals, which randomly couple to form the stable lignin polymer (Ralph *et al.*, 2004; Vanholme *et al.*, 2010). Plant class III peroxidases are secreted iron-containing enzymes that use hydrogen peroxide to produce substrate radicalization (Marjamaa *et al.*, 2009; Shigeto and Tsutsumi, 2016). In Arabidopsis there are 73 peroxidase genes annotated in the genome (Welinder *et al.*, 2002). Specific peroxidases have been demonstrated to be required for lignin polymerization under particular developmental contexts, such as the inflorescence stem (Shigeto *et al.*, 2013) and in lignification of the root Casparian strip (Lee *et al.*, 2013). Laccases are heavily glycosylated, multi-copper-containing oxidative enzymes that use a copper oxidation center to strip electrons from monolignols, thereby generating monolignol radicals; the Arabidopsis laccase family consists of 17 genes with diverse expression patterns, including in lignifying cells (Turlapati *et al.*, 2011). Berthet *et al.* (2011) demonstrated the roles of Arabidopsis LACCASE4 (LAC4) and LACCASE17 (LAC17) in stem lignification through double-mutant *lac4/lac17* studies. Chemical analysis and cross-sections of stems in *lac4/lac7* double-mutants showed decreased lignin content and collapsed xylem vessels that were unable to withstand the negative pressure of water transport. Triple *lac4/lac17/lac11* Arabidopsis mutants demonstrated that loss of these laccases causes plants to be dwarf with little lignin (Zhao *et al.*, 2013). Despite elevated peroxidase gene expression, the number of lignified cells in the triple *lac4/lac17/lac11* mutants was shown to be greatly decreased in both the root and the stem (Zhao *et al.*, 2013).

Oxidative enzymes are therefore very important to the process of lignification, yet surprisingly little is known about how these cell wall proteins are arranged and held in the secondary cell wall. In previous studies, we showed that LAC4 and LAC17 were localized to secondary cell wall domains, and were responsible for the helical deposition of lignin in protoxylem tracheary elements (TEs) (Schuetz *et al.*, 2014). When red fluorescent protein (mCHERRY)-tagged LAC4 was expressed in the *lac4/lac17* double-mutant, driven by its native promoter (*proLAC4:LAC4-mCHERRY*), the collapsed xylem phenotype in the whole plant was rescued, indicating that the LAC4-mCHERRY was functional (Schuetz *et al.*, 2014). Little is known about localization of peroxidases involved in lignification. Nevertheless, *PEROXIDASE64* (PRX64) was expressed in the endodermis during root development and it was localized to the Casparian strip when fused to the mCHERRY protein (Lee *et al.*, 2013). Although no *prx64* mutants have been isolated in Arabidopsis, plants with microRNA-mediated knockdown of PRX64 displayed delays in lignification of the Casparian strip, confirming a role in lignification of the root endodermis (Lee *et al.*, 2013).

In this study, we investigate the localization and mobility of PRX64 and LAC4 in two secondary cell wall model systems in *Arabidopsis thaliana*: the lignifying inflorescence stem and ectopically produced protoxylem tracheary elements. Protoxylem TE formation was induced using a plant line that expresses *VASCULAR NAC DOMAIN 7* (*VND7*), a NAC domain transcription factor that triggers protoxylem vessel cell fate (Kubo *et al.*, 2005), fused to an inducible glucocorticoid receptor (*VND7-GR*; Yamaguchi *et al.*, 2010). In stems, the mCHERRY-tagged PRX64 was expressed only in fibers, and was exclusively localized in the middle lamella/cell corners. In contrast, LAC4-mCHERRY was expressed in all cells that have thickened secondary cell walls, i.e. vessels and fibers, and it was highly immobile in the secondary cell wall. These data indicate that LAC4 is strongly cross-linked into the secondary cell wall once secreted, and this provides a mechanism to direct the spatial control of lignification.

## Materials and methods

### Plant growth

*Arabidopsis thaliana* plants were grown in growth chambers (Conviron) with 16/8 h (light/dark) photoperiod at a constant 21 °C. Surface-sterilized seeds were plated on half-strength Murashige and Skoog (MS) medium (PhytoTechnology Laboratories) and vernalized at 4 °C for 2–3 d before being transferred to the growth chambers. Seedlings were transferred to soil 7 d after germination. Seedlings harbouring the *pro35S:VND7-VP16-GR* construct for ectopic protoxylem formation were plated on GM media (MS media supplemented with 1% sucrose and 1× Gamborg’s Vitamin Mix), and induced with 10 μM dexamethasone (DEX) in half-strength liquid MS media as described by Watanabe *et al.* (2015). Cross-sections from mature inflorescence stems of Arabidopsis were generated by hand-sectioning using fresh razor blades on stems in a drop of water on parafilm under a dissecting microscope.

All transgenic plant lines were generated using ecotype Columbia-0 of *Arabidopsis thaliana* plants, *Agrobacterium tumefaciens* (strain GV3101), and the floral dip method (Clough and Bent, 1998). The *proPRX64:PRX64-mCHERRY* (Lee *et al.*, 2013) line was obtained from Niko Geldner (University of Lausanne). The *irx10 –/–, irx10L +/–* line was obtained from Simon Turner (University of Manchester), and they were transformed with the *pro35S:VND7-VP16-GR* construct (Yamaguchi *et al.*, 2010). The *proLAC4:LAC4-mCHERRY* construct (Schuetz *et al.*, 2014) was transformed into *pro35S:VND7-VP16-GR irx10 –/–, irx10L +/–* plants, and those that were segregating *irx10 –/–, irx10L –/–, pro35S:VND7-VP16-GR proLAC4:LAC4-mCHERRY* genotypes were isolated in later generations and used in fluorescence recovery after photobleaching (FRAP) experiments.

### Microscopy

A Leica DMR epifluorescence microscope was used to image lignin autofluorescence and mCHERRY (excitation 340–380 nm and emission 450–500 nm, and excitation 520–580 nm and long-pass emission filter 560+ nm, respectively). A Perkin-Elmer UltraView VoX spinning disk confocal mounted on a Leica DMI6000 inverted microscope and a Hamamatsu 9100–02 CCD camera were used for high-resolution localization of mCHERRY-tagged proteins (excitation 561 nm, emission 595–625 nm) and lignin autofluorescence (excitation 405 nm, emission 440–510 nm). Using this set-up, FRAP measurements and analyses were done using the Volocity FRAP plug-in. For each FRAP measurement, six pre-bleach images were obtained, and a square region of interest (ROI) of 1.5 µm was bleached (561-nm laser at 100% intensity). Post-bleach images were taken at maximum speed either at (1) one image per second for 60 or 120 s, or (2) one image per 20–30 s for 300 s. Similar to Martinière *et al.* (2011), first-order diffusion kinetics were observed, indicating freely diffusing fluorophores. FRAP recovery curves were fitted using a single exponential equation [*f*(*t*) = *y* + *Ae*^–*kt*^; where *t* = time, *y* = mobile fraction, *A* = mobile fraction with bleach correction, and *k* = fitting parameter of the curve. The half-time of recovery (*T*_½_), describing the time from bleach until 50% of the final recovered fluorescence was reached, was calculated as *T*_½_=ln0.5/*k*. All statistical comparisons for average mobile fractions were performed using the Mann–Whitney non-parametric *U*-test, run using the GraphPad™ Prism® software. Although identical laser settings were used, the periphery of the bleached area tended to be more diffuse in the primary cell wall than the secondary cell wall. This might create a potential bias towards lower recovery intensities in the primary cell wall (Martinière *et al.*, 2011), but in fact the opposite trend was observed (see Results).

### Inhibitor treatments

Arabidopsis seedlings at 3–5 d old were treated with either piperonylic acid (PA) to inhibit cinnamate-4-hydroxylase in the general phenylpropanoid pathway (Schalk *et al.*, 1998) and hence to decrease lignin production (Kaneda *et al.*, 2008), or the cellulose inhibitor 2,6-dichlorobenzonitrile (DCB), which inhibits cellulose synthase (Meyer and Herth, 1978; Debolt *et al.*, 2007). *proLAC4:LAC4-mCHERRY* and *proUBQ10:sec-mCHERRY* seeds were grown in the dark for 3–5 d on GM agar (0.75%) plates and transferred to 24-well culture plates containing half-MS media. For lignin inhibition, seedlings were incubated with 10 μM PA (in DMSO) in the dark for 6 h at 21 °C, after which DEX was added into the wells and the plates were returned to 21 °C for 36 h. The seedlings were then mounted in liquid half-MS for imaging. For cellulose inhibition, seedlings were incubated with 10 μM DCB (2,6-dichlorobenzonitrile dissolved in DMSO) and 10 μM DEX. Culture plates were returned to 21 °C for 36 h, and the seedlings were then mounted in liquid half-MS for imaging.

To ensure that the PA was effective in inhibiting lignin deposition under these experimental conditions, PA-treated and mock-treated seedlings were mounted in half-MS media and imaged for lignin autofluorescence under ultraviolet light (excitation 340–380 nm) using a Leica DMR compound microscope equipped with a EBQ 100 Isolated Mercury Lamp. Images were captured using the Canon EOS Rebel T5 and EOS Utility software. DCB-treated and mock-treated seedlings were stained for cellulose in 16.5mg ml^–1^ Direct Red 23 (Sigma-Aldrich) for 30 min and washed twice in half-MS solution. Seedlings were mounted in half-MS and imaged using a Perkin-Elmer UltraView VoX spinning disk confocal mounted with a Hamamatsu 9100–02 CCD camera (excitation 561 nm, emission 595–625 nm). Mock and treated seedlings were imaged using identical microscope settings. Images were processed using ImageJ (Schneider *et al.*, 2012) and hypocotyl cells were analysed for corrected fluorescence as described by Smith *et al.* (2017). A Welch’s two-sample *t*-test for unequal variances was performed using R (www.r-project.org). Comparisons of PA and mock used data from 15–18 seedlings for each treatment (*t*=5.8379, d.f.=17.818, *P*=1.638 × 10^–5^), and comparisons of DCB and mock used data from 45–48 stained induced cells from15–16 seedlings for each treatment (*t*=4.7777, d.f.=74.769, *P*=8.641 × 10^–6^).

## Results

Inflorescence stems of Arabidopsis are a useful experimental system in which to study secondary cell walls and lignification, as gene expression, cell wall composition, and development have been characterized in detail (Hall and Ellis, 2013). Comparing gene expression of all peroxidase (PRX) and laccase (LAC) gene family members from data provided by previous studies gave a snapshot of which PRX and LAC genes are most abundant in lignifying cells of Arabidopsis inflorescences (see Supplementary Fig. S1 at *JXB* online). PRX64 (encoded by AT5G42180) is by far the most highly expressed peroxidase in the lignifying stem, and its promoter activity has been demonstrated in this organ (Tokunaga *et al.*, 2005; Smith *et al.*, 2017). LAC4 (encoded by AT2G38080) is one of the most highly expressed laccases in the stem, along with LAC17 and LAC11 (Supplementary Fig. S1; Berthet *et al.*, 2011; Zhao *et al.*, 2013). Interestingly, the LAC genes but not PRX64 were strongly co-expressed with CESA4, 7, and 8, and several other genes that have important functions during secondary cell wall biosynthesis (Supplementary Fig. S2). These expression patterns support the role of laccases in lignification of secondary cell walls, and in addition lend evidence to the hypothesis that *PRX64* may have multiple roles, as it has a different gene co-expression network compared with LAC4. From this gene expression data, we chose LAC4 and PRX64 for further investigation.

**Fig. 1.**
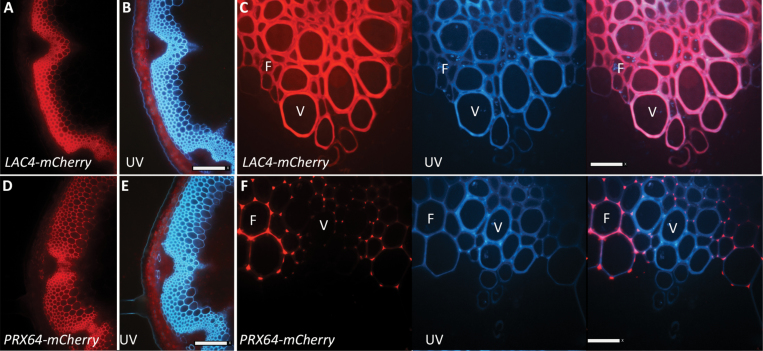
Distinct cell-type distributions of lignin-related oxidative enzymes LAC4 and PRX64 in Arabidopsis stems. (A) Epifluorescence of a transverse section of stems from *proLAC4:LAC4-mCHERRY* plants, with red fluorescence in all secondary cell walls. (B) Blue UV autofluorescence from lignin of the section shown in (A). (C) Spinning disk confocal image of one vascular bundle from a LAC4-mCHERRY stem with red fluorescent protein (RFP) fluorescence in both vessels (V) and fibers (F). UV autofluorescence shows lignin, and the overlap of UV-RFP is shown in the merged image on the right. (D) Epifluorescence of a transverse section of stems from *proPRX64:PRX64-mCHERRY* plants, with red fluorescence in secondary cell walls of fibers. (E) Blue UV autofluorescence from lignin of the section shown in (D). (F) Spinning disk confocal image of one vascular bundle of from PRX64-mCHERRY plants with RFP fluorescence only in fibers (F), but not vessels (V). UV autofluorescence shows lignin, and the overlap of UV-RFP is shown in the merged image on the right. All plants were 8–9 weeks old, and sampled at the base of mature (25–30 cm) inflorescence stems. Scale bars: (A,B,D,E) = 1 mm; (C,F) = 40 μm.

In order to examine the detailed spatial distribution of the highly expressed LAC4 in the inflorescence stem of Arabidopsis, transverse sections were prepared from plant lines expressing functional *proLAC4:LAC4-mCHERRY* in the *lac4/lac17* mutant background (Schuetz *et al.*, 2014), and in all samples, LAC4-mCHERRY localized to secondary cell wall domains. Epifluorescence of whole-stem transverse sections of *proLAC4:LAC4-mCHERRY* showed the red mCHERRY signal in vascular bundles and interfascicular fibers (Fig. 1A), and the merged blue lignin autofluorescence indicated that LAC4 was found in all lignified cells (Fig. 1B). Control wild-type stems without fluorescent protein showed red chlorophyll autofluorescence in non-lignifying photosynthetic cortical cells, and blue lignin autofluorescence in vascular bundles and extra-xylary interfascicular fibers (see Supplementary Fig. S3). Using confocal microscopy, the detailed distribution of LAC4 in the secondary cell walls of xylem vessels, xylem fibers, and interfascicular fibers was revealed, with strong co-localization of the red LAC4-mCHERRY signal and the blue lignin autofluorescence in the merged image (Fig. 1C).

In contrast to the broad distribution of LAC4, PRX64 expression, demonstrated by fluorescently tagged PRX64 driven by its native promoter (*proPRX64:PRX64-mCHERRY*), was observed only in fibers: both interfascicular fibers between vascular bundles and in the xylary fibers (Fig. 1D). PRX64 was not observed in xylem vessels (Fig. 1D, F), although the vessels were clearly marked by lignin autofluorescence (Fig. 1E, F). This is consistent with gene expression data from *VND7-GR* plant lines ectopically induced to form protoxylem vessels, where *PRX64* expression was absent during vessel differentiation, whereas LAC4 was highly expressed (Yamaguchi *et al.*, 2010, 2011; Supplementary Table S1). Confocal microscopy was used to examine details of PRX64-mCHERRY localization in the vascular bundle, and showed that the red PRX64-mCHERRRY signal was present in the xylem, but it was restricted to xylary fibers. The blue lignin autofluorescence was observed in xylem vessels that lacked PRX64-mCHERRY, as seen in the merged image (Fig. 1F). Thus, PRX64 was only found in a subset of the cells in the lignifying inflorescence stem and did not localize to xylem vessels, in contrast to LAC4, which was localized to the secondary cell walls in all lignified cells.

**Fig. 2.**
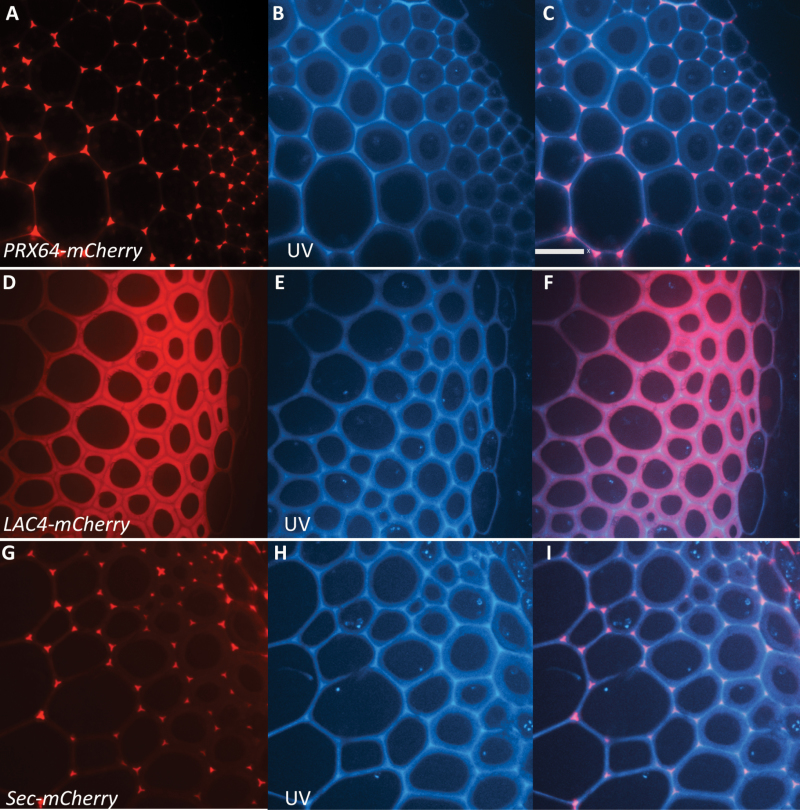
The lignin-related oxidative enzymes LAC4 and PRX64 are found in different secondary cell wall domains in Arabidopsis stem fibers. (A) Spinning disk confocal images of interfascicular fibers from stems of proPRX64:PRX64-mCHERRY Arabidopsis plants with red fluorescence protein (RFP) fluorescence only in the middle lamella and cell corners. (B) UV autofluorescence shows lignin. (C) Overlap of UV and RFP in a merged image. (D) Spinning disk confocal images of interfascicular fibers from stems of *proLAC4:LAC4-mCHERRY* Arabidopsis plants with RFP fluorescence in secondary walls but not the middle lamella or cell corners. (E) UV autofluorescence shows lignin. (F) Overlap of UV and RFP in a merged image. (G) Spinning disk confocal images of interfascicular fibers from stems of control small secreted red fluorescent protein (sec-mCHERRY) in Arabidopsis plants with RFP fluorescence mainly in the middle lamella and cell corners. (H) UV autofluorescence shows lignin. (I) Overlap of UV and RFP in a merged image. All plants were 8–9 weeks old, and sampled at the base of mature (25–30 cm) inflorescence stems. Scale bars =40 μm.

Since both PRX64 and LAC4 were localized to interfascicular fibers, confocal microscopy was used to detail their distributions in the thick secondary cell walls of these cells. Surprisingly, the two oxidative enzymes had contrasting localization patterns: PRX64 was localized to the cell corners and the middle lamella, the part of the cell wall with the brightest lignin autofluorescence (Fig. 2A–C), whereas LAC4 was not present in the cell corners or the middle lamella but was found throughout the thick secondary cell wall (Fig. 2D–F). Thus, in sclerenchyma cells and xylary fibers, these two oxidative enzymes each occupied a different cell wall domain, with LAC4 exclusively in the thick secondary cell wall and the PRX64 concentrating in the cell corners and the middle lamella between cells.

In order to evaluate the localization of a non-oxidative enzyme protein in these cell types, a small secreted red fluorescent protein (sec-mCHERRY) was engineered using the N-terminal signal sequence from a lipid transfer protein gene (LTPG; DeBono *et al.*, 2009) fused to mCHERRY, driven by the constitutively expressed *Ubiquitin10* (*UBQ10*) gene promoter. sec-mCHERRY was predicted to be targeted to the endoplasmic reticulum due to its N-terminal signal sequence, then to follow a default secretion pathway to the cell wall (Denecke *et al.*, 1990; Batoko *et al.*, 2000). sec-mCHERRY was efficiently secreted out of cells and showed a similar pattern of protein accumulation in the cell corners and the middle lamella as PRX64-mCHERRY (Fig. 2G–I).

**Fig. 3.**
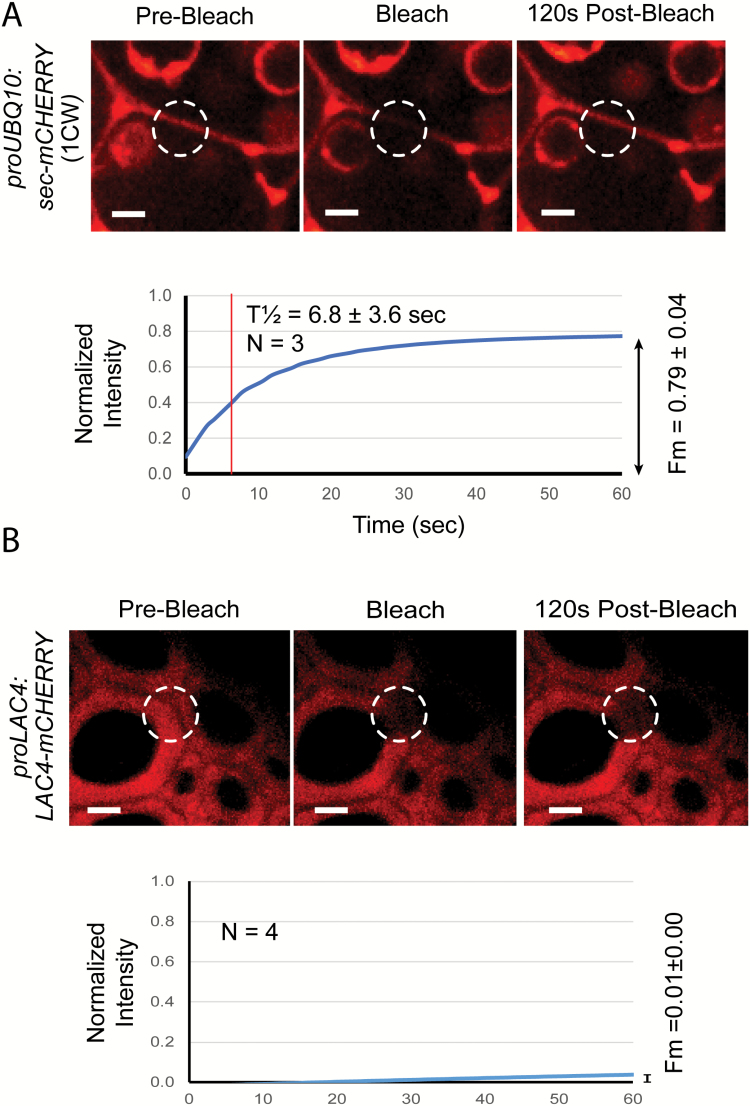
Fluorescence recovery after photobleaching (FRAP) of cell wall proteins in Arabidopsis stems. (A) Mobility of a small secreted control protein (sec-mCHERRY) in primary cell walls of pith cells. (B) Lack of mobility of the laccase LAC4 in fiber secondary cell walls. All experiments were performed with fresh hand-sections of the base of mature (25–30 cm) stems from 8-9-week-old plants. Scale bars = 3 μm.

In order to test the mobility of proteins in the cell wall, fluorescence recovery after photobleaching (FRAP) was used on stem transverse sections. FRAP experiments were performed by photobleaching fluorescence in a specified region of interest (ROI), then measuring the mobility of neighbouring fluorescent proteins as they moved into the ROI, generating recovery curves from the normalized fluorescence intensity data. The fraction of recovered fluorescence over the initial fluorescence (mobile fraction, *F*_m_) as well as the time (in seconds) taken to reach half of the overall fluorescence recovered (*T*_½_) was calculated. To test the cell wall FRAP system, sec-mCHERRY was bleached in a ROI of the primary cell wall, and recovery of a high mobile fraction was observed (*F*_m_=0.79 ± 0.04) as the sec-mCHERRY rapidly repopulated the primary cell wall (*T*_½_=6.8 ± 3.6 s) (Fig. 3A).

The FRAP system was used to test the mobility of the LAC4-mCHERRY in the secondary cell walls of fibers, and it was found to be highly immobile (*F*_m_=0.01 ± 0.004), and full recovery of fluorescence in the ROI was not observed (Fig. 3B). The lack of recovery of fluorescence indicates that LAC4 is strongly cross-linked into the secondary cell wall matrix. It was not possible to target a control protein to the thick layers of secondary cell wall where LAC4 was found, as the control sec-mCHERRY did not remain in the S1, S2, or S3 layers, but accumulated in the cell corners of fibers in a similar pattern to PRX64 (Fig. 2G–I). However, when FRAP was applied to the lignified cell corners of fibers, both sec-mCHERRY and PRX64 were also immobile (see Supplementary Fig. S4).

**Fig. 4.**
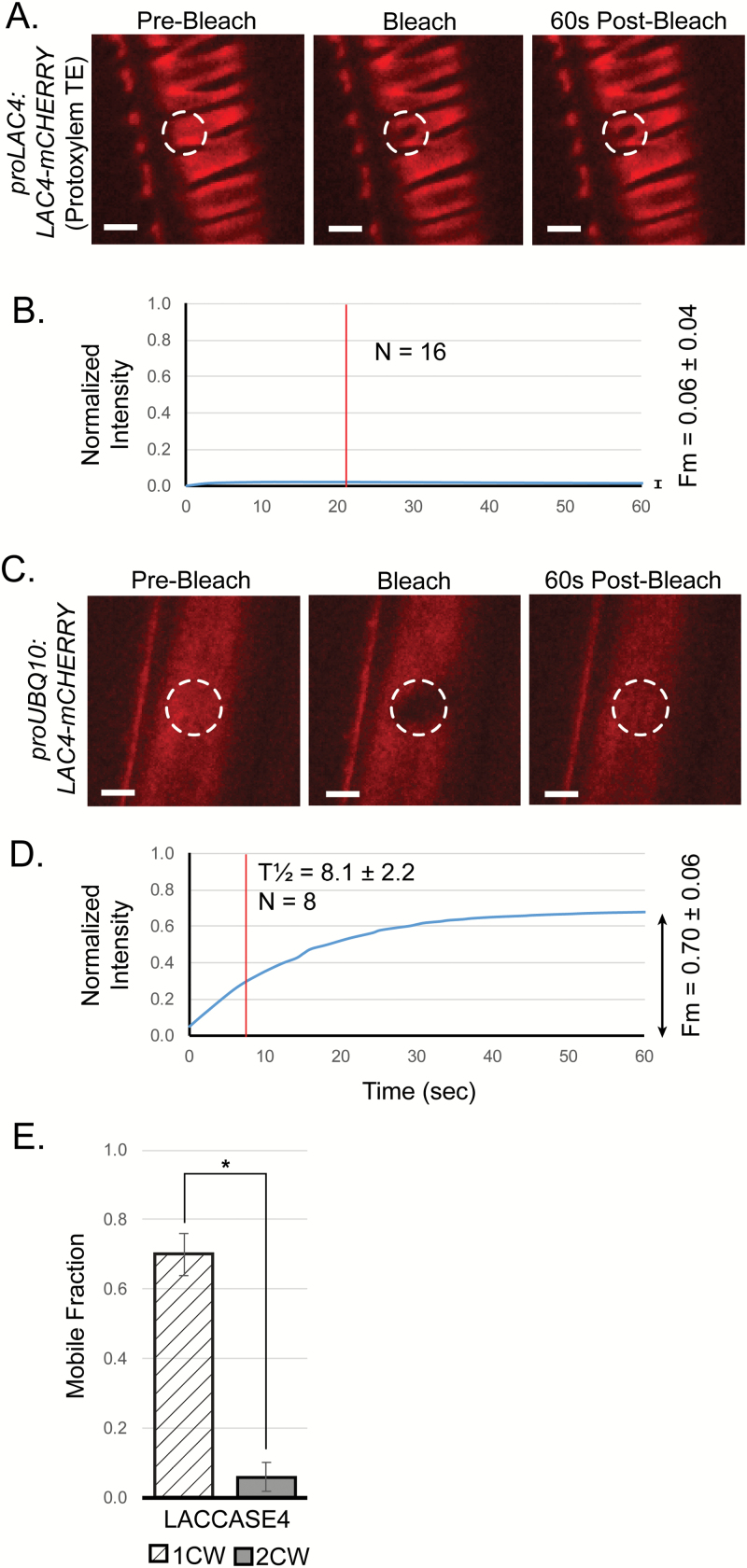
Laccases are immobile in the secondary cell wall of induced protoxylem tracheary elements (TEs). (A) LAC4-mCHERRY in secondary cell walls of apical hook cells of *proLAC4:LAC4-mCHERRY/VND7-GR* Arabidopsis seedlings 36 h after dexamethasone (DEX) induction. Left, pre-bleaching of the region of interest (ROI, white dashed circle); centre, at the time of bleaching; right, 60 s after bleaching. (B) Fluorescence recovery after photobleaching (FRAP) recovery curve for the sample shown in (A), showing the mobile fraction (*F*_m_) and *T*_½_ (vertical line). (C) LAC4-mCHERRY ectopically expressed in primary cell walls in *proUBQ10:LAC4-mCHERRY/VND7-GR* lines without DEX induction. Left, pre-bleaching the of ROI (white dashed circle); centre, at the time of bleaching; right, 60 s after bleaching. (D) FRAP recovery curve of the sample shown in (C), showing a large mobile fraction. (E) The mobility (*F*_m_) of the mCHERRY-tagged laccase in the secondary cell walls (2CW) was significantly lower than in the primary cell walls (1CW) (Mann–Whitney *U*-test, *P≤0.0001). Data are means ±SD. Scale bars = 3 μm.

To verify the finding that LAC4 was remarkably immobile in the secondary cell wall, we used Arabidopsis transgenic plants that expressed *VASCULAR NAC DOMAIN 7* (*VND7*), the NAC domain transcription factor that triggers protoxylem vessel cell fate (Kubo *et al.*, 2005), fused to an inducible glucocorticoid receptor system (*GR*) (Yamaguchi *et al.*, 2010). Upon addition of the glucocorticoid DEX, plants transformed with both *VND7-GR* and *proLAC4:LAC4-mCHERRY* have been shown to produce abundant LAC4-mCHERRY in secondary cell wall domains (Schuetz *et al.*, 2014). Use of the native promoter ensured that LAC4-mCHERRY was only expressed during secondary cell wall production, and we observed LAC4-mCHERRY in secondary, but not primary, cell walls (Fig. 4A). As with the interfascicular fibers of the stem, when FRAP was applied to the protoxylem secondary cell walls, the mobility of LAC4-mCHERRY was very low, and the ROI appeared permanently bleached (Fig. 4A). It did not recover fluorescence even after allowing recovery for 300 s after bleaching (see Supplementary Fig. S5).

To test whether LAC4 is generally immobile, even in a primary cell wall, LAC4-mCHERRY was expressed ectopically in primary cell walls of seedlings using a constitutive promoter (*proUBQ10:LAC4-mCHERRY*) (Fig. 4C) in the *VND7-GR* background but in absence of DEX, when no ectopic secondary cell wall was made. Ectopically expressed LAC4-mCHERRY in the primary cell wall showed fluorescence that recovered quickly post-bleach through diffusion (Fig. 4D). Comparing primary (no DEX) and secondary cell walls (following induction by DEX), a significantly larger mobile fraction of fluorescently tagged LAC4-mCHERRY migrated into the ROI in the primary cell wall (*F*_m_=0.70 ± 0.106, *n*=8 cells) than the very limited mobile fraction of LAC4-mCHERRY in the secondary cell wall (Fig. 4B, *F*_m_=0.06 ± 0.04, *n*=16 cells). In addition, the mean half-time of recovery of LAC4-mCHERRY in the primary cell wall (*T*_½_=8.1 ± 2 s) was faster than in the secondary cell wall (*T*_½_=21 ± 25 s). Thus, laccases were significantly more mobile in the primary than in the secondary cell wall (Fig. 4E).

**Fig. 5.**
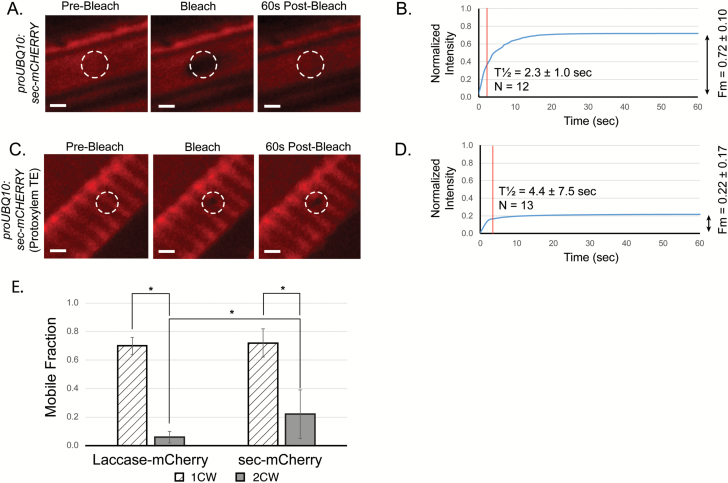
Secreted mCHERRY (sec-mCHERRY) is less mobile in the secondary cell wall than the primary cell wall. (A, C) Fluorescence recovery after photobleaching (FRAP) images taken of apical hook cells of Arabidopsis seedlings containing *proUBQ10:sec-mCHERRY*/*VND7-GR* pre-bleaching the region of interest (ROI, white dashed circles), at the time of bleaching, and 60 s after bleaching. (A) Rapid recovery of sec-mCHERRY in uninduced seedlings with primary cell walls. Scale bars = 3 μm. (B) The mobile fraction (*F*_m_) and *T*_½_ (vertical line) were calculated for the FRAP experiments done on the uninduced cells shown in (A). (C) Slow recovery of sec-mCHERRY in the secondary cell walls of protoxylem tracheary element cells, 36 h after induction of *VND7-GR*. (D) The mobile fraction (*F*_m_) was calculated for the FRAP experiments done on the cells shown in (C). (E) Comparison of LAC4-mCHERRY and sec-mCHERRY mobility in primary cell walls (1CW) and secondary cell walls (2CW). Within 2CW environments sec-mCHERRY had statistically higher recovery than LAC4-mCHERRY (Mann–Whitney *U*-test, *P*≤0.05); however, the mobility of both LAC4-mCHERRY and sec-mCHERRY were much greater in 1CW than 2CW (Mann–Whitney *U*-test, *P≤0.0001). Data are means ±SD.

To test whether the difference observed was due to a general difference in the primary and secondary cell wall environments, the mobility of the control secreted protein in the two cell walls was tested. Plants containing both the sec-mCHERRY protein construct (*proUBQ10:sec-mCHERRY*) and the inducible *VND7-GR* construct were examined prior to protoxylem cell fate induction, and the sec-mCHERRY was uniformly and diffusely found throughout the primary cell wall (Fig. 5A). When these cells were tested for sec-mCHERRY mobility in the primary cell wall, rapid recovery of a high mobile fraction was observed (Fig. 5B). In contrast, the sec-mCHERRY fluorescence recovery was slower in secondary cell walls of protoxylem TE cells (Fig. 5C), and displayed limited recovery (Fig. 5D). The mean half-time of recovery (*T*_½_) was also longer for secreted mCHERRY in the secondary cell wall (*T*_½_=4.4 ± 7.5) than in the primary cell wall (*T*_½_=2.3 ± 1.0). These fluorescence recovery patterns demonstrate that a small, secreted mCHERRY protein was significantly more mobile in primary than in secondary cell wall environments (Fig. 5B, D), suggesting that differences in cell wall composition or organization can play an important role in protein mobility in the cell wall.

Despite both the laccase and small sec-mCHERRY control protein having a similar overall pattern of mobility in the two different cell wall environments, there were some interesting differences. Whereas the fluorescence recovery of laccase and sec-mCHERRY in the primary cell wall was similar, LAC4-mCHERRY had a significantly lower mobile fraction than sec-mCHERRY in the secondary cell wall environment (Fig. 5E).

**Fig. 6.**
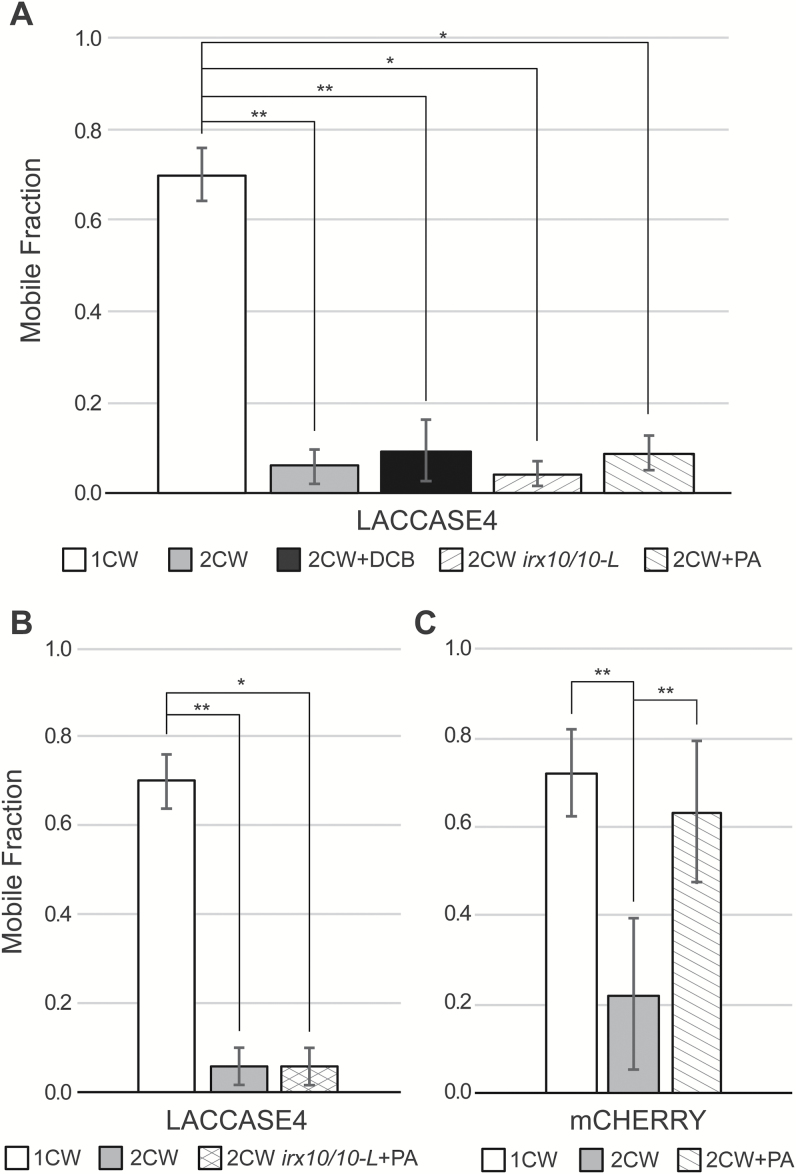
Laccases are highly immobile in the secondary cell wall. Fluorescence recovery after photobleaching (FRAP) in the primary cell walls (1CW) and secondary cell walls (2CW) of seedlings carrying *proLAC4:LAC4-mCHERRY/VND7-GR* (LACCASE4) or proUBQ*10:sec-mCHERRY/VND7-GR* (sec-mCHERRY). (A) Decreased levels of cellulose (10 μM 2,6-dichlorobenzonitrile, DCB, for 36 h), xylan (*irx10/irx10-L* mutants), or lignin (10 µM piperonylic acid, PA) did not change the low mobility of laccases. (B) FRAP mobile fraction when xylan and lignin were simultaneously disrupted using *irx10/irx10-L* mutants transformed with *proLAC4:LAC4-mCHERRY/VND7-GR* and treated with PA. (C) Although 1CW sec-mCHERRY mobility was higher than 2CW mobility, the lack of recovery in 2CW was reversed when lignin biosynthesis was inhibited with PA (Mann–Whitney *U*-test, **P≤0.0001, *P≤0.001). Data are means ±SD.

Since LAC4 was relatively immobile in the secondary cell wall domains of protoxylem TEs compared to primary cell walls, the different composition or organization of the components that make up the secondary cell wall could be hindering protein mobility. Candidates for components that bind laccases to the secondary cell wall are the major hemicellulose glucuronoxylan (xylan), secondary cell wall cellulose, or the phenolic polymer lignin. To examine the effects of reduced xylan abundance on LAC4-mCHERRY mobility, the *proLAC4:LAC4-mCHERRY* construct was transformed into a xylan-deficient double-mutant *irregular xylem 10/irregular xylem10-like* (*irx10/10-L*; Brown *et al.*, 2005) in the *VND7-GR* background. The herbicide 2,6-dichlorobenzonitrile (DCB) was used to inhibit cellulose production (Meyer and Herth, 1978; Debolt *et al.*, 2007). Control experiments demonstrated that DCB treatment inhibited cellulose biosynthesis and deposition in the secondary cell walls of *VND7-GR*-induced seedlings (see Supplementary Fig. S6). To ascertain what happens to laccase mobility in the secondary cell wall with reduced lignin, Arabidopsis seedlings transformed with the construct *proLAC4:LAC4-mCHERRY* in the *VND7-GR* background were treated with piperonylic acid (PA), an inhibitor of cinnamate-4-hydroxylase, a key enzyme in the general phenylpropanoid pathway required for lignin production (Schalk *et al.*, 1998; Kaneda *et al.*, 2008). PA-treated *VND7-GR*-induced seedlings showed significantly decreased lignin autofluorescence in secondary cell walls compared to mock-treated seedlings (Supplementary Fig. S7). FRAP was conducted on ectopic protoxylem TE secondary cell walls, and, in all treatments and mutants, the fluorescence recovery of the LAC4-mCHERRY in seedlings was not different from the lack of recovery in wild-type control secondary cell walls (Fig. 6A). Combining xylan-deficient mutants (*irx10/irx10-L*) with PA to inhibit lignin formation in the xylan-deficient mutants produced similar results (Fig. 6B). These data suggest that no single element of the secondary cell wall anchors the laccase to the wall, or that eliminating components of the secondary cell wall leads to a collapsed wall architecture, as suggested by (Paës *et al.*, 2017).

To test whether the lack of mobility of laccases in lignin-deficient secondary cell walls is specific to LAC4-mCHERRY, or if it is generally true of other secreted proteins, plant lines containing sec-mCHERRY (*proUBQ10:sec-mCHERRY*) in the *VND7-GR* background were also treated with 10 µM PA, and FRAP was conducted on PA-treated protoxylem TEs (Fig. 6C). FRAP analysis of sec-mCHERRY in secondary cell walls treated with PA showed higher recovered fluorescence intensity (*F*_m_=0.63 ± 0.16) than that of sec-mCHERRY in the secondary cell walls of control protoxylem TEs (*F*_m_=0.22 ± 0.17). Together, this data suggests that lignin imposes general constraints on the mobility of proteins in the secondary cell wall, as seen by the increased mobility and of sec-mCHERRY, but that LAC4 is especially constrained in its mobility.

## Discussion

The aim of this study was to explore how oxidative enzymes are arranged and held in the secondary cell wall. Both *PEROXIDASE64* (PRX64) and *LACCASE4* (LAC4) are highly expressed in the lignifying inflorescence stem of Arabidopsis and have known roles in lignification. LAC4 was found in all lignifying cell types, while PRX64 was only in fibers. Within the fiber secondary cell wall, there were distinctive and mutually exclusive localization patterns, with PRX64 in cell corners and the middle lamella and LAC4 throughout the thick secondary cell wall layers. Theoretically, these patterns could be generated during fiber development by the exocytosis of peroxidase-rich vesicles to the cell wall early in development followed by laccase secretion, or potentially by mobility of proteins in the wall following secretion. However, the movement of fluorescently tagged cell wall proteins had not been studied *in planta*. Therefore, we used FRAP to examine the oxidative enzyme mobility in Arabidopsis stems and in induced protoxylem tracheary elements (TEs). PRX64, as well as the small secreted control protein, sec-mCHERRY, were immobile in cell corners and the middle lamella of secondary cell walls of the stem. In the stems, LAC4-mCHERRY was also tightly anchored in the secondary cell wall environment. Because LAC4 was more widely expressed, including in vessels, we were able to do a more detailed characterization using FRAP to quantify the dynamic behaviour of this laccase within secondary cell wall environments of inducible protoxylem TEs. Using the inducible system and a constitutive promoter, we could contrast LAC4 and a control protein, sec-mCHERRY, in both primary and secondary wall environments. The mobility of both proteins was always higher in the primary than the secondary cell wall, although the sec-mCHERRY control was more mobile than LAC4 in the secondary wall environment.

In the Arabidopsis stem, distinctive tissue-specific protein expression patterns were found for PRX64 and LAC4. *PRX64* expression was restricted to xylary, interfascicular, and phloem fibers (Fig. 1). As fibers are rich in S-lignin, and previously characterized peroxidase loss-of-function mutants (*prx2*, *prx25*, *prx71*, *prx72*) show phenotypes with altered S/G ratios (Herrero *et al.*, 2013; Shigeto *et al.*, 2013, 2015), this result is consistent with a role for peroxidases in S-lignin formation in fibers. In contrast, *LAC4* was expressed in all lignifying cells including xylem vessels. This result is consistent with the collapsed-vessel phenotype that led to the identification of the *lac4* mutant allele as an irregular xylem (*irx12*) mutant (Brown *et al.*, 2005). In addition, high expression of the *LAC4* gene was reported upon activation of VND7, the master regulator for xylem vessel differentiation (Yamaguchi *et al.*, 2011). The wide distribution of LAC4 in all lignifying cell types is probably related to the dramatic loss of lignin in *lac4 lac11 lac17* triple-mutants (Zhao *et al.*, 2013). It will be interesting to see if this pattern of PRX in fibers only, compared to LAC in all lignifying cell types, holds true to other members of these extensive gene families.

Not only did PRX64 and LAC4 have distinctive cell types, they were also found in different cell wall domains in the fibers (Fig. 2). LAC4-mCHERRY localized to the thick secondary wall layers, whereas PRX64-mCHERRY localization was concentrated in the cell corners and the middle lamella between cells. This spatial separation may provide a rationale for the severe reductions in lignin deposition that have been observed in laccase mutants, because thick secondary cell walls represent more of the biomass than the relatively thin primary middle lamella. Moreover, histochemical lignin staining in laccase mutants suggests that the middle lamella and cell corners still lignify in contrast to a severe reduction in secondary cell wall lignification (Berthet *et al.*, 2011; Zhao *et al.*, 2013). These data support the notion that different protein localization patterns could impact the lignification of specific cell wall domains.

Laccases play important roles in directing lignin deposition, as evidenced by mutant phenotypes in Arabidopsis inflorescence stems (Berthet *et al.*, 2011; Zhao *et al.*, 2013) and in the spiral secondary cell walls of protoxylem TEs (Schuetz *et al.*, 2014). In order to evaluate the post-secretion mobility of LAC4 in the secondary cell wall, we utilized both Arabidopsis stem sections and the VND7-inducible protoxylem system, and performed FRAP analysis of LAC4-mCHERRY in the primary and secondary cell walls. In both experimental systems, the mobility of LAC4-mCHERRY in the secondary cell wall was tightly constrained (Figs 3, 4). Use of the VND7-GR system permitted us to express the control protein, sec-mCHERRY, in the secondary cell wall thickenings as well (Fig. 5), and this showed that the composition and/or structure of the secondary cell wall constrains protein movement generally. However, LAC4 was more constrained within the secondary cell wall than sec-mCHERRY. Laccases are heavily glycosylated proteins, with LAC4 itself having 14 predicted N-glycosylation sites (Strong and Claus, 2011; Turlapati *et al.*, 2011), making it possible that the oligosaccharide chains may retard the movement of this glycoprotein within the polysaccharide matrix. It is also possible that LAC4 may be immobilized by an unknown specific component of secondary cell walls found only in cells where LAC4 is normally expressed, e.g. a protein co-expressed with LAC4 or a fiber/vessel-specific cell wall epitope.

Little is known about how the cell wall matrix influences the movement of proteins, although estimates of the size of a protein that can diffuse into primary cell wall fragments range from 17 kDa (Carpita *et al.*, 1979) to 60–67 kDa (Baron-Epel *et al.*, 1988). Although LAC4 is predicted to be 61 kDa in size, and is highly glycosylated, it was still mobile when ectopically expressed in primary cell walls. In this study, the lateral diffusion of both LAC4-mCHERRY and the control protein sec-mCHERRY was faster in the primary cell wall compared to the secondary cell wall. If the primary cell wall is organized in such a way that allows for constant expansion of the wall, and it is highly hydrated, then there may be more ‘free space’ for proteins such as laccases or a secreted protein to move within its matrix. In the secondary cell wall, lignin cross-bridging to hemicelluloses associated with abundant cellulose microfibrils may block these spaces, which is probably why the mobility of sec-mCHERRY increased in secondary cell walls when the plants were treated with the monolignol inhibitor piperonylic acid (Fig. 6). Recent bioenergy studies of the penetration of carbohydrate-active enzymes, such as cellulases, indicate that the cell wall porosity represents an important parameter in accessibility of enzymes for digestion (reviewed by Meng and Ragauskas, 2014). These studies may provide insight as to why perturbation of the secondary cell wall components did not affect LAC4 mobility, as loss of lignin is hypothesized to lead to collapse of the cellulose/hemicellulose network (Paës *et al.*, 2017)

In summary, fluorescently tagged enzymes and confocal microscopy revealed the arrangement of oxidative enzymes in the secondary cell walls of Arabidopsis stems and inducible protoxylem TEs. In fibers, LAC4 was embedded in the xylan-rich secondary cell wall layers of both vessels and fibers, while PRX64 was in the cell corners and the middle lamella of fibers only. LAC4 had very limited mobility in secondary cell walls in stems and in induced protoxylem TEs, even following disruption of components of the secondary cell wall. These data suggest that LAC4 may be co-secreted with the glucuronoxylans of the secondary cell wall, which is consistent with their co-expression with genes such as *IRX9* and *IRX10*. It may also be enmeshed in the abundant cellulose microfibrils of the secondary cell wall, as it remained immobile after removal of both xylan and lignin. The firm anchoring of LAC4 to the secondary cell wall can provide strong spatial segregation into specific cell wall domains, providing a mechanism for directing localized lignification.

## Supplementary data

Supplementary data are available at JXB online.

Fig. S1. Relative expression of peroxidase and laccase genes in Arabidopsis.

Fig. S2. Relationship among co-expressed gene networks of *LAC4*, *PRX64*, and secondary cell wall *CESA*.

Fig. S3. Autofluorescence of Arabidopsis wild-type stem.

Fig. S4. Lack of fluorescence recovery after photobleaching of cell wall proteins in secondary cell walls of Arabidopsis stems.

Fig. S5. Laccases are immobile in the secondary cell wall of protoxylem tracheary elements, even after an extended recovery period.

Fig. S6. 2,6-dichlorobenzonitrile treatment interferes with cellulose biosynthesis in secondary wall bands of induced *VND7-GR* seedlings.

Fig. S7. Piperonylic acid treatment inhibits lignin biosynthesis and deposition in secondary walls of induced *VND7-GR* seedlings.

Table S1. Gene expression data of oxidative enzymes in lignifying Arabidopsis models, for VND7-induced tracheary elements and Arabidopsis stems.

Supplementary Figure Table

## Author contributions

MS, YW, NH, ALS, and EYC planned and designed the experiments; MS, YW, NH, and EYC conducted the experiments; EYC, NH, RS conducted the data analysis and statistics; all authors contributed to the writing of the manuscript.
