# Supplementary material for: Distribution, mobility, and anchoring of lignin-related oxidative enzymes in Arabidopsis secondary cell walls
Source: J Exp Bot. 2018 Feb 22;69(8):1849–59. doi: 10.1093/jxb/ery067 (PMC6018803; doi:10.1093/jxb/ery067)
Supplement: Supplementary Figure Table [file ery067_suppl_supplementary_figure_table.pdf]

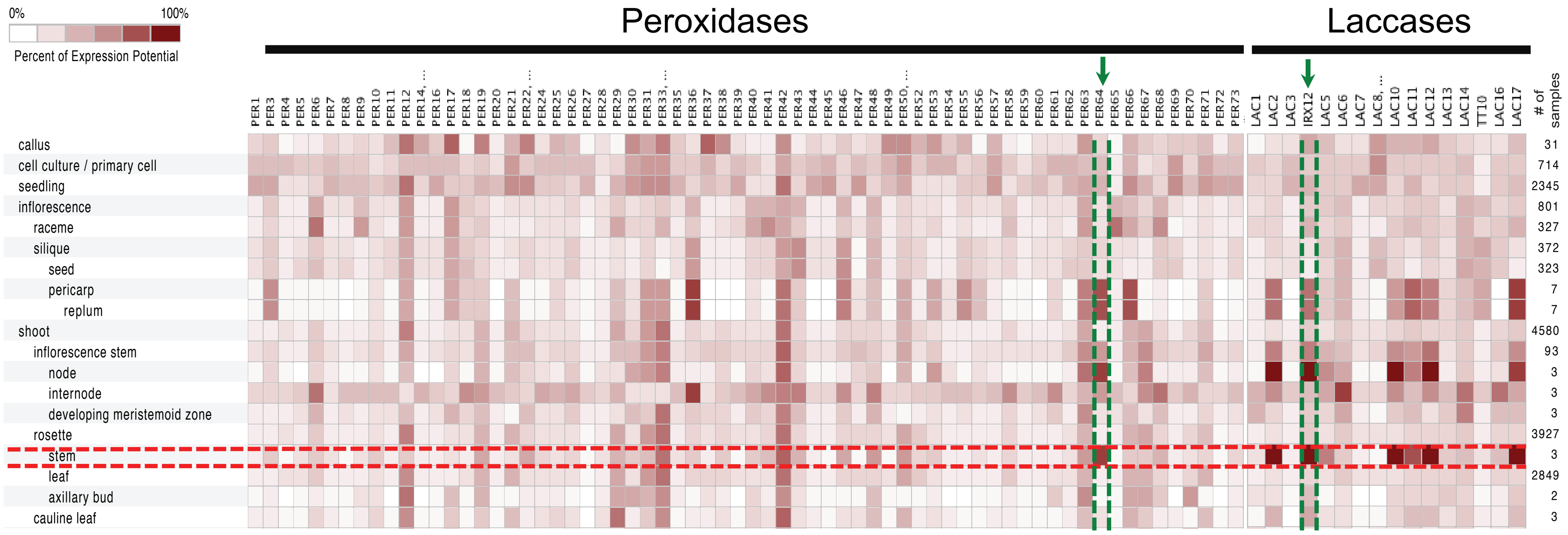

Fig. S1: Relative expression of peroxidase and laccase genes in Arabidopsis

Heat map was preformed with Genevestiagtor (http://genevestigator.com; Hruz *et al*., 2008). Relative transcrpit levels of Arabidopsis peroxidase and laccase genes are compared in selected tissue. AT_AFFY_ATH1-0 chip was used for data analysis. Doted lines highlight specifice expression patterns of LAC4 (IRX12; AT2G38080) and PRX64 (PER64; AT5G42180). PRX64 is the most highly expressed peroxidase in inflorescence stem. While LAC4, LAC2, LAC10, LAC12 and LAC17 are the most expressed.

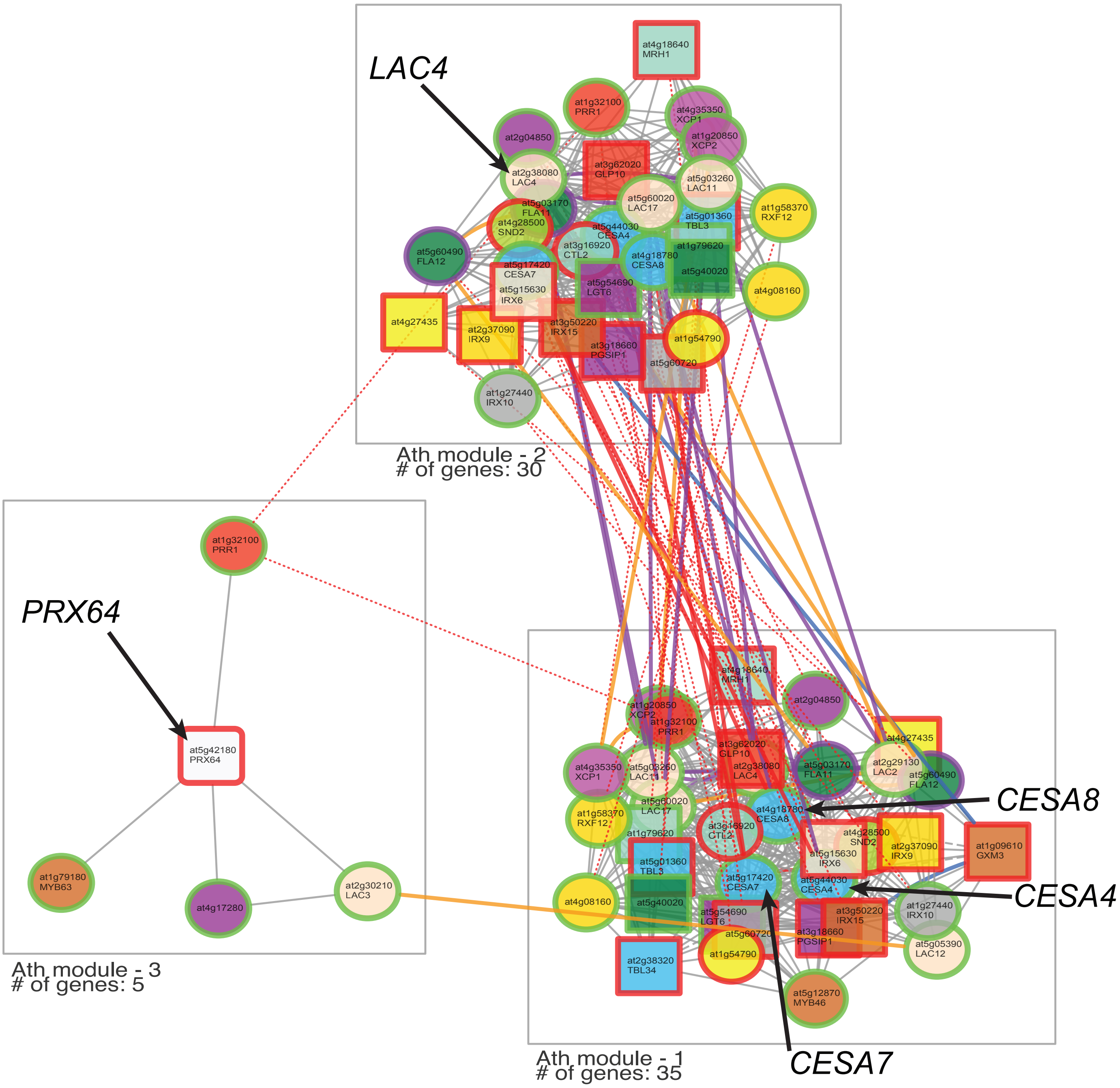

Fig. S2: Relationship between coexpressed gene networks of LAC4, PRX64 and secondary cell wall CESAs.

Gene co-expression networks were generated with the Network-comparer tool of PlaNet (http://aranet.mpimp-golm.mpg.de). Network comparer (Ruprech *et al.*, 2016) was used to find close relationships between co-expression networks of Arabidopsis LAC4 (AT2G38080), PRX64 (AT5G42180) and CESA4, -7, and -8 (AT5G44030, AT5G17420, AT4G18780). The three boxes represent gene modules, while nodes found in the modules represent genes. The colored shapes of the nodes indicates genes that belong to the same gene families or contain same Pfam domains. For the analysis, secondary cell wall CESAs were assigned to same group (module) whereas LAC4 and PRX64 were assigned to two different groups. There is a clear relationship between LAC4 and secondary cell wall CESA coexpression networks but not with PRX64.

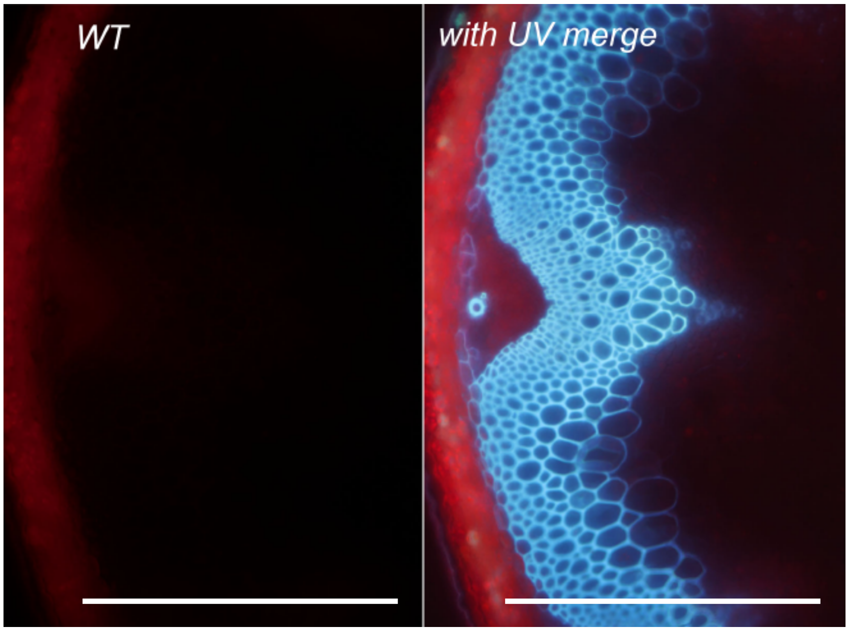

Fig. S3: Auto-fluorescence of Arabidopsis *wild-type* stem, transverse cross section viewed with epi-fluorescence.
Left, red fluorescent channel with chlorophyll autofluorescence. Right, merged image of red channels with blue autofluorescence of lignin produced by UV-excitation. 8-9 week-old plants were sectioned at the base of mature 25-30 cm inflorescence stems. Scale bar =1 mm.

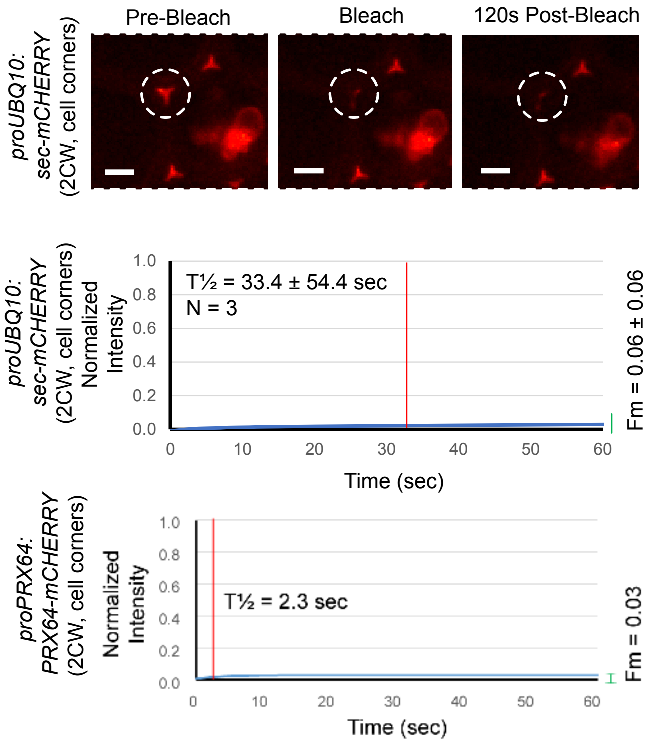

Fig. S4. Lack of Fluorescence Recovery After Photobleaching (FRAP) of cell wall proteins in secondary cell walls of Arabidopsis stems.

Mobility of a small secreted control protein (sec-RFP) in cell corners of interfascicular fibers, and lack of mobility of PRX64-mCHERRY in fiber secondary cell walls. All experiments were performed with fresh hand sections of the base of mature 25-30 cm stems. Scale bars = 3 µm.

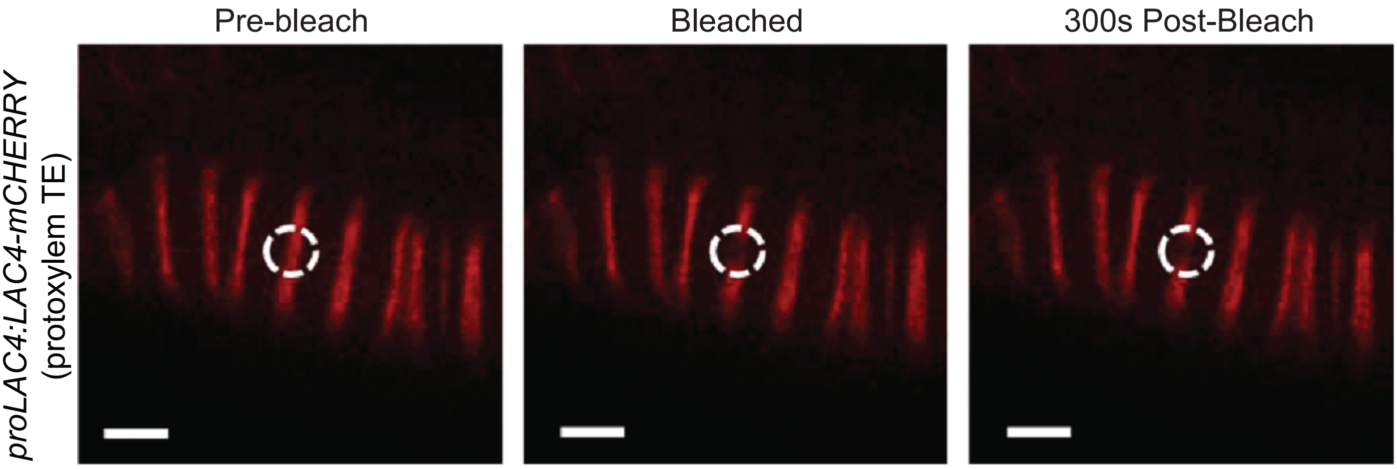

Fig. S5. Laccases are immobile in the secondary cell wall of protoxylem tracheary elements. Fluorescence recovery after photobleaching (FRAP) was preformed on VND7-GR induced Arabidopsis etiolated-etiolated seedlings containing *proLAC4:LAC4-mCHERRY*. Images were taken before bleaching, at the time of bleaching, and 300 sec after bleaching. RFP-tagged laccase fluorescence does not recover even with the extended time course. Scale bars = 3 μm.

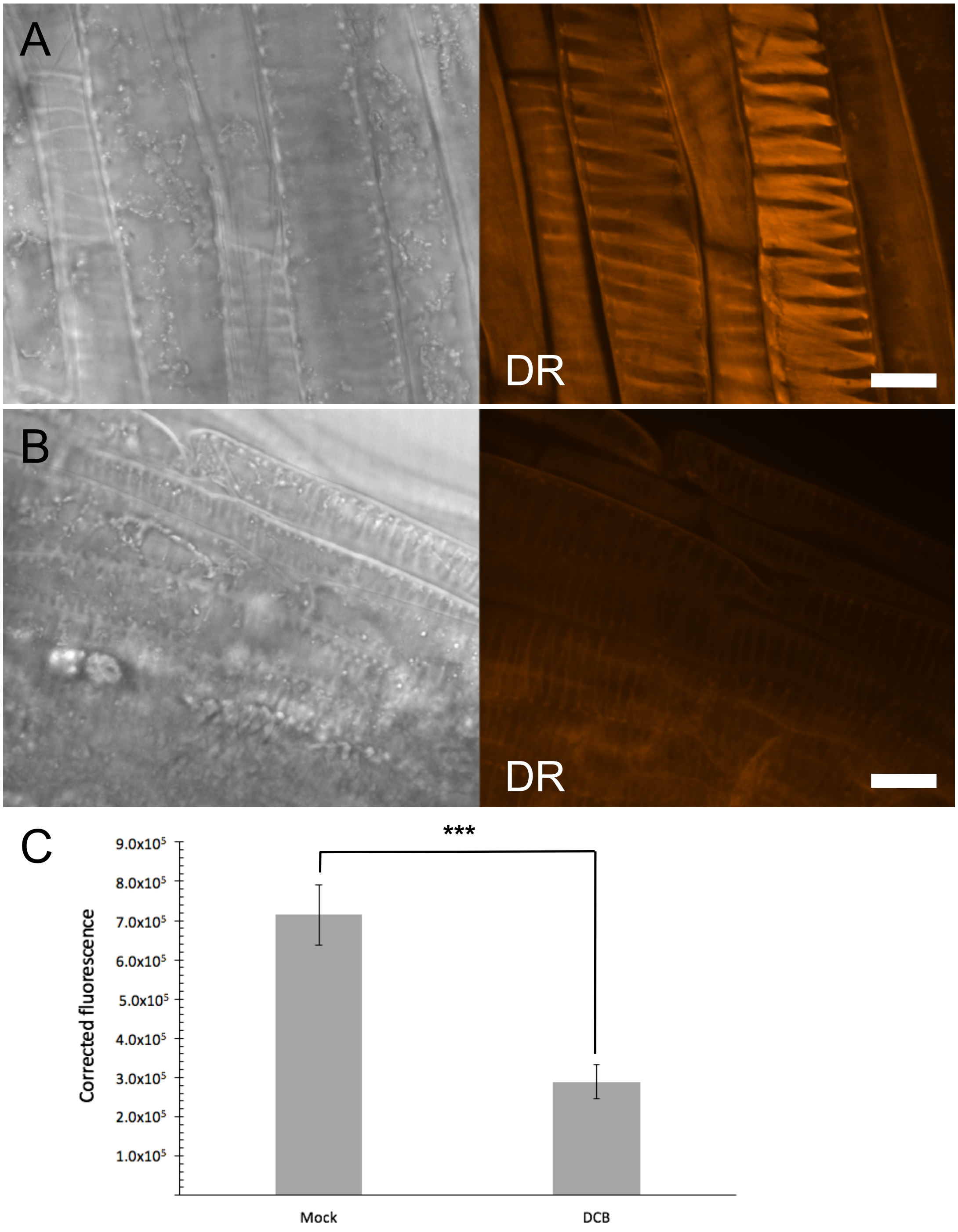

Fig. S6: 2,6-dichlorobenzonitrile (DCB) treatment interferes with cellulose biosynthesis and secretion in secondary wall bands of induced *VND7-GR* seedlings.
(A) Mock-treated *VND7-GR* seedlings stained with direct red 23 (DR; orange) show normal protoxylem secondary cell wall banding following induction. (B) DCB-treated seedlings show reduced and abnormal cell wall cellulose deposition in secondary cell walls. Scale bars = 16μm. (C) Bar graph of average corrected autofluorescence of mock- and DCB-treated *VND7-GR* induced seedlings, n = 45-48 unduced cells per treatment, ***$p < 0.001$. Bars = *SE*.

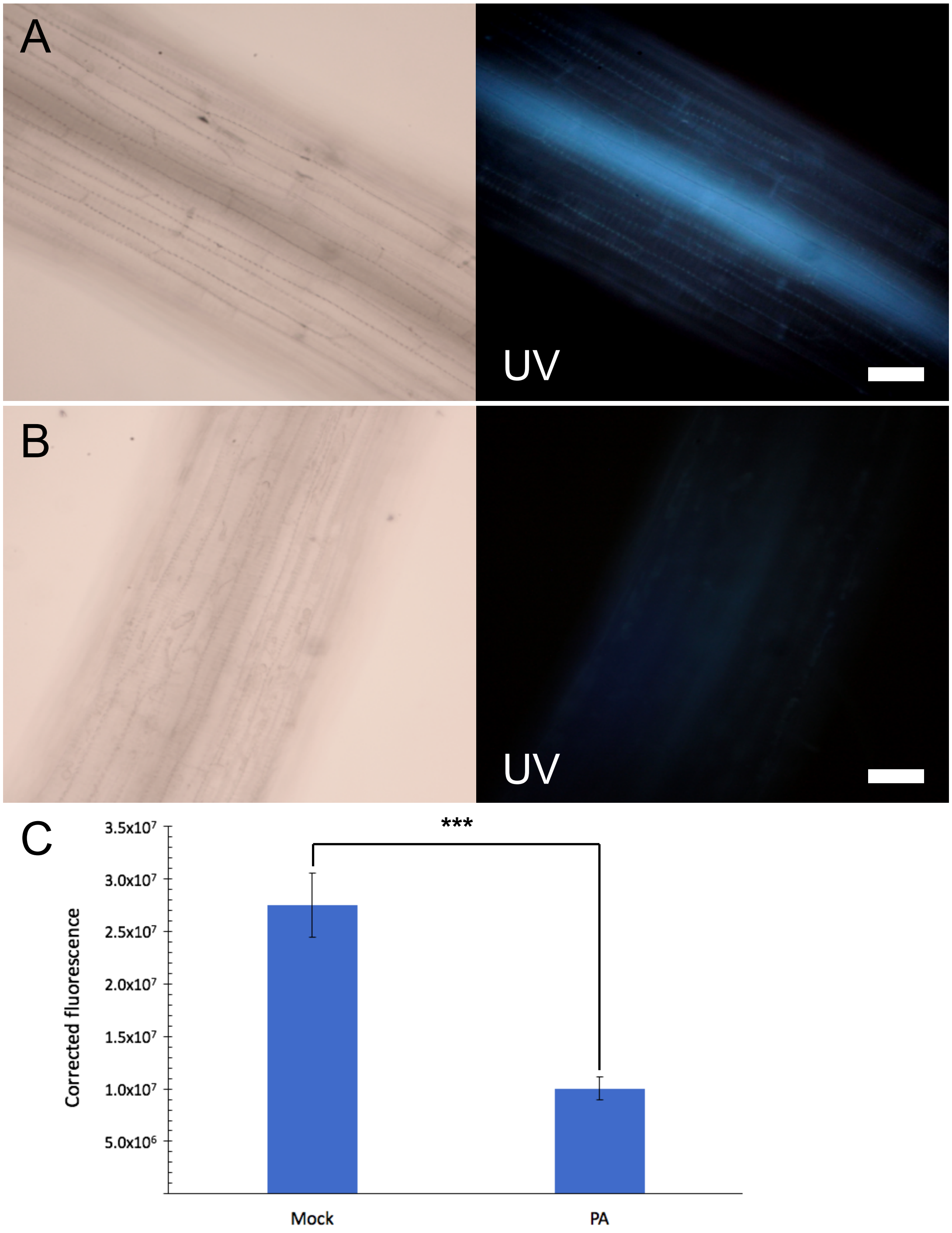

Fig. S7: Piperonylic acid (PA) treatment inhibits lignin biosynthesis and deposition in secondary walls of induced *VND7-GR* seedlings.
(A) Mock-treated *VND7-GR* seedlings show lignin autofluorescence (blue) in protoxylem bands following induction. (B) PA-treated *VND7-GR* seedlings show highly reduced autofluorescence in secondary cell walls following induction. Scale bars = 50µm. (C) Bar graph of average corrected autofluoresence of mock- and PA-treated *VND7-GR* induced seedlings, n = 15-18 seedlings/treatment, *** p <0.001. Bars = SE.

Table S1: Gene expression data of oxidative enzymes in lignifying Arabidopsis models, either VND7-induced tracheary elements (Yamaguchi et al., 2011) or Arabidopsis stems (Hall and Ellis, 2013)

data from Yamaguchi et al., 2011

Gene ID	Gene Name	Gene expression of Arabidopsis cultured cells differentiating into tracheary elements				
		2day/0day	4day/0day	6day/0day	8day/0day	10day/0day
AT5G60020	Laccase 17	1.0	238.5	858.4	491.2	273.1
AT2G38080	Laccase 4	0.7	95.1	241.6	181.2	111.1
AT5G03260	Laccase 11	1.1	8.1	88.7	55.3	24.1
AT5G51890	Peroxidase 66	2.1	12.7	73.6	14.8	7.5
AT4G36430	Peroxidase 49	14.2	26.3	16.0	13.5	11.2
AT1G71695	Peroxidase 12	0.2	0.3	4.8	3.1	1.7
AT2G18140	Peroxidase 14	9.8	4.9	4.5	3.6	4.0

data from Hall and Ellis, 2013

Gene ID	Gene Name	Gene expression of stem base / stem tip
AT2G38080.1	Laccase 4	23.9
AT2G29130.1	Laccase 2	5.5
AT5G05390.1	Laccase 12	4.8
AT5G60020.1	Laccase 17	2.8
AT2G40370.1	Laccase 5	2.3
AT5G42180.1	Peroxidase 64	40.9
AT2G37130.1	Peroxidase 21	14.8
AT4G37520.1	Peroxidase 50	6.4
AT4G08780.1	Peroxidase 38	6.0
AT4G08770.1	Peroxidase 37	2.4
AT5G47000.1	Peroxidase 65	2.4
AT5G19890.1	Peroxidase 59	2.3
AT2G18140.1	Peroxidase 14	2.2
